# Patients’ Experiences of Telephone-Based and Web-Based Cognitive Behavioral Therapy for Irritable Bowel Syndrome: Longitudinal Qualitative Study

**DOI:** 10.2196/18691

**Published:** 2020-11-20

**Authors:** Stephanie Hughes, Alice Sibelli, Hazel A Everitt, Rona Moss-Morris, Trudie Chalder, J Matthew Harvey, Andrea Vas Falcao, Sabine Landau, Gilly O'Reilly, Sula Windgassen, Rachel Holland, Paul Little, Paul McCrone, Kimberley Goldsmith, Nicholas Coleman, Robert Logan, Felicity L Bishop

**Affiliations:** 1 Centre for Clinical and Community Applications of Health Psychology Department of Psychology University of Southampton Southampton United Kingdom; 2 Department of Biostatistics and Health Informatics Institute of Psychiatry, Psychology and Neuroscience King’s College London London United Kingdom; 3 Academic Department of Psychological Medicine Institute of Psychiatry, Psychology and Neuroscience King’s College London London United Kingdom; 4 Department of Gastroenterology Southampton University Hospital Southampton United Kingdom; 5 Kings College Hospital London United Kingdom

**Keywords:** irritable bowel syndrome, cognitive behavioral therapy, internet, primary health care, self-management

## Abstract

**Background:**

Cognitive behavioral therapy (CBT) is recommended in guidelines for people with refractory irritable bowel syndrome (IBS). However, the availability of CBT is limited, and poor adherence has been reported in face-to-face CBT.

**Objective:**

Nested within a randomized controlled trial of telephone- and web-delivered CBT for refractory IBS, this qualitative study aims to identify barriers to and facilitators of engagement over time with the interventions, identify social and psychological processes of change, and provide insight into trial results.

**Methods:**

A longitudinal qualitative study was nested in a randomized controlled trial. Repeated semistructured interviews were conducted at 3 (n=34) and 12 months (n=25) post baseline. Participants received telephone-based CBT (TCBT; n=17 at 3 months and n=13 at 12 months) or web-based CBT (WCBT; n=17 at 3 months and n=12 at 12 months). Inductive thematic analysis was used to analyze the data.

**Results:**

Participants viewed CBT as credible for IBS, perceived their therapists as knowledgeable and supportive, and liked the flexibility of web-based and telephone-based delivery; these factors facilitated engagement. Potential barriers to engagement in both groups (mostly overcome by our participants) included initial skepticism and concerns about the biopsychosocial nature of CBT, initial concerns about telephone-delivered talking therapy, challenges of maintaining motivation and self-discipline given already busy lives, and finding nothing new in the WCBT (WCBT group only). Participants described helpful changes in their understanding of IBS, attitudes toward IBS, ability to recognize IBS patterns, and IBS-related behaviors. Consistent with the trial results, participants described lasting positive effects on their symptoms, work, and social lives. Reasons and remedies for some attenuation of effects were identified.

**Conclusions:**

Both TCBT and WCBT for IBS were positively received and had lasting positive impacts on participants’ understanding of IBS, IBS-related behaviors, symptoms, and quality of life. These forms of CBT may broaden access to CBT for IBS.

## Introduction

### Background

Irritable bowel syndrome (IBS) affects 10%-20% of the general population [1]. Official UK guidelines for the management of IBS [1] recommend provision of diet and lifestyle advice, a trial of medications, and—if patients have ongoing troublesome symptoms after 12 months (refractory IBS)—referral for psychological intervention, such as cognitive behavioral therapy (CBT). CBT can improve IBS symptom severity and quality of life [2-5]. However, barriers to CBT for IBS exist, including limited availability of face-to-face CBT for IBS, uncertain cost-effectiveness [6], and issues with poor adherence [7]. The Assessing Cognitive behavioral Therapy in Irritable Bowel (ACTIB) trial [8] aimed to determine the clinical effectiveness and cost-effectiveness of therapist-delivered, telephone-based CBT (TCBT) and web-based CBT (WCBT) for IBS. Both TCBT and WCBT groups showed significant improvements in IBS symptoms at 12 months, compared with treatment as usual (TAU) [9,10]. Scores from the IBS Symptom Severity Scale [11] were 61.6 (95% CI 33.8-89.5) points lower (*P*<.001) in TCBT and 35.2 (95% CI 12.6-57.8) points lower (*P*=.002) in WCBT at 12 months, than TAU.

Therapist-delivered TCBT and WCBT may overcome some of the barriers to traditional face-to-face CBT by offering better cost-effectiveness for health care commissioners [12] and providing greater flexibility in timing and location for patients [9]. Although some people may prefer remote access therapies, it may not be appropriate for all patients. Providing remote treatment options can increase access and free up more intensive face-to-face resources for those patients for whom remote intervention is not appropriate. Patients’ experiences of these modalities of CBT for IBS have rarely been studied and could provide novel insights into the processes underpinning treatment uptake, adherence, and effectiveness.

We previously explored patients’ experiences of using WCBT as part of a feasibility trial of the prototype of the WCBT program used in the ACTIB trial [13]. Participants in that study were positive about WCBT and described the website as “well designed and easy to understand and use,” although some felt that “a user had to be self-motivated to work through the material.” Participants engaged with the website to varying degrees, with some having limited or no engagement because “they did not find the website relevant to them” or “the website was too impersonal.” Follow-up was performed at just 12 weeks, so experiences of longer-term effects could not be assessed. To the best of our knowledge, this is the only study on patients’ experiences of TCBT and WCBT for IBS. Studies in other populations suggest that WCBT is acceptable and helpful and allows a level of anonymity when disclosing personal thoughts [14]. Similarly, trials of TCBT in other populations have shown encouraging results in terms of symptom improvement, with no detrimental impact on patient satisfaction [15].

### Objectives

We conducted a large qualitative study nested within the ACTIB trial. Previously reported analyses using this data set have focused on treatment seeking and appraisal processes [16], patients’ perspectives on general practitioner (GP) interactions [17], and emotional processing in IBS [18]. The aim of this study is to explore patients’ experiences and views of TCBT and WCBT for IBS immediately posttreatment and at 12-month follow-up. The objectives are to identify factors that facilitate or impede engagement with web-delivered and telephone-delivered CBT in this patient group both during and after the main intervention period, to identify social and psychological processes of change in the short term and long term, and to provide insight into the quantitative results of this complex trial.

## Methods

### The ACTIB Trial and Interventions

The ACTIB trial recruited 558 participants from primary care (GP) and secondary care (gastroenterology clinics) in Southampton and London between March 2014 and March 2016. The participants were randomized to one of the following 3 groups: TCBT, WCBT, and TAU. The TCBT group received six 1-hour TCBT sessions over 9 weeks, a detailed patient manual, and 2 *booster* 60-min follow-up phone calls at 4 and 8 months. The WCBT group received access to the previously piloted IBS digital self-management program *Regul8* [4,8]. Regul8 consisted of 8 web-based sessions to be completed on a weekly basis, three 30-min telephone support sessions over 9 weeks, and 2 *booster* 30-min follow-up phone calls at 4 and 8 months. The CBT content delivered via telephone in the TCBT arm and via website in the WCBT arm was the same, with only the mode of delivery being different. Both intervention groups also received ongoing TAU, in primary or secondary care or both, as appropriate. The 2 interventions contained similar content, and the same therapists provided telephone support. The CBT content was based on an empirical cognitive behavioral model of IBS [19] and comprised education and behavioral and cognitive techniques aimed at improving bowel habits, developing stable healthy eating patterns, addressing unhelpful thoughts, managing stress, reducing symptom focusing, and preventing relapse [8]. The TAU group continued with their usual care (in primary care or secondary care or both, as appropriate) and were offered access to Regul8 on completion of the trial. For further details, see the trial protocol [8]. The study was approved by the relevant National Research Ethics Service Committee on June 11, 2013 (13/SC/0206).

### Nested Qualitative Study

#### Design

A longitudinal qualitative study was nested within the ACTIB trial in an embedded mixed methods design with the qualitative component acting in a supportive capacity [20]. Repeated, also known as serial, semistructured interviews were conducted with the same participants at 3 and 12 months. Serial interviews are rarely used in medical research but, compared with one-off interviews, are better suited to exploring patients’ experiences over time and changes therein [21]. Therefore, we chose serial interviews because our objectives were oriented toward processes that occur in time (eg, identifying processes of change in the trial) and because we were interested in how patients’ experiences and reflections might change from the initial therapy phase to the subsequent follow-up phase.

#### Data Collection

Purposeful sampling was used to select a range of ACTIB participants to invite for interview. To best address our objectives and to capture the experiences of a diverse range of individuals, we sought to interview participants from all 3 ACTIB groups and to include variation within each group in gender, age, ethnic background, geographical location (Southampton or London), symptom severity score, and recruitment path (primary or secondary care). Of 558 participants, 100 were invited to take part in an interview, 58 of whom agreed to participate. The data for this analysis comprised the interviews conducted with people from the TCBT and WCBT groups at 3 months postbaseline (n=34) and at 12 months postbaseline (n=24). The characteristics of these participants are summarized in Table 1, demonstrating the breadth of our sample.

**Table 1:** Baseline demographic and clinical characteristics of interviewees by trial group.

Characteristics		Therapist CBT^a^		Web-based CBT			Total sample		
		3 months (n=17)	12 months (n=12)		3 months (n=17)	12 months (n=12)		3 months (n=34)	12 months (n=24)
Gender (female), n (%)		13 (76)	10 (83)		14 (82)	9 (75)		27 (79)	19 (79)
**Ethnicity, n (%)**									
	White British	11 (65)	7 (58)		12 (71)	9 (75)		23 (68)	16 (67)
	White other	4 (24)	4 (33)		4 (24)	3 (25)		8 (24)	7 (29)
	Mixed White and Asian	1 (6)	1 (8)		0	0		1 (3)	1 (4)
	African	1 (6)	0		0	0		1 (3)	0
	Other ethnicity	0	0		1 (6)	0		1 (3)	0
Age (years), mean (SD)		39.94 (11.71)	38.4 (10.4)		42.41 (17.37)	45 (18.63)		41.18 (14.64)	41.7 (15.14)
Irritable Bowel Syndrome Severity Scoring System baseline score, mean (SD)		283.47 (117.11)	278.58 (126.07)		259.65 (124.39)	219.58 (123.01)		271.56 (119.57)	249.08 (125.35)
**Recruitment site, n (%)**									
	Primary care	11 (65)	8 (67)		13 (76)	9 (75)		24 (71)	17 (71)
	Secondary care	6 (35)	4 (33)		4 (24)	3 (25)		10 (29)	7 (29)
Duration of symptoms in years before study entry, mean (SD)		14.71 (7.10)	12.83 (6.94)		15.59 (8.89)	16.33 (9.24)		15.15 (7.93)	14.58 (8.19)
Length of irritable bowel syndrome diagnosis when entering the trial, mean (SD)		7.94 (7.66)	6.5 (6.53)		11.82 (9.22)	13 (8.72)		9.88 (8.59)	9.75 (8.23)

^a^CBT: cognitive behavioral theory.

Interviews were conducted either face-to-face (n=9) or via telephone (n=49), lasted between 22 and 113 min, and were audio-recorded and transcribed verbatim using unique participant identification numbers to preserve anonymity and permit linkage between repeated interviews. A semistructured topic guide was used flexibly, allowing the interviewer to explore any relevant issues raised by the participants. The topic guides for the 3-month and 12-month interviews are available in Multimedia Appendices 1 and 2, respectively. The topic guides included open-ended questions on expectations about the ACTIB trial and reasons for taking part, previous experiences of IBS therapies and management, experiences of being in the trial and the allocated therapy, and any changes that occurred since starting the trial. Interviews at 3 months continued until data saturation, that is, the point at which no new themes relevant to the research questions were identified. This was reached when 34 participants had been interviewed. The same 34 participants were contacted again at 12 months. Of 34 participants, 24 agreed to be interviewed again. The remaining 10 either failed to respond or declined, citing a lack of time to take part. Data saturation for themes related to posttrial experiences, and longer-term retrospective reflections on trial experiences was reached within those 24 interviews, making additional recruitment unnecessary.

#### Data Analysis

Interviews were read repeatedly before being coded in NVivo (QSR International; version 11) and analyzed by working iteratively with the phases mapped out by Braun and Clarke for inductive thematic analysis [22] supplemented with techniques from grounded theory (Table 2) [23,24].

**Table 2:** Summary of the analytic process.

Thematic analysis phase	Implementation	Supplementary techniques derived from grounded theory
Familiarization	Initial notes made as transcripts read repeatedly	Listen to audio recordings
Generate initial codes	Using the first 22 transcripts, initial codes and a coding manual were developed. This coding manual was used to analyze subsequent transcripts, and amendments were made iteratively when necessary	Line-by-line open coding on a portion of the data and constant comparison
Searching for themes	As the analysis evolved, codes related to similar manifest or latent concepts were grouped together. These groupings were considered as candidate themes and subthemes	Constant comparison, identify key concepts in the data, and write memos
Reviewing themes	Candidate themes and subthemes were reviewed to ensure that they worked in relation to the coded extracts and the individual interviews and that they captured relevant material from across the data set	Constant comparison, search for deviant cases, generate selected case summaries to capture participant stories, and changes across 3- and 12-month interviews
Defining and naming themes and their interrelations	Themes were refined and explicitly defined to clearly and succinctly capture patterns in the data relevant to the research objectives Cross-tabulations (using NVivo’s matrix query) to compare theme content and relevance between the TCBT^a^ and WCBT^b^ groups and between 3 and 12 months	Constant comparison
Reporting	Selected compelling examples to illustrate themes and subthemes. Final analysis and contextualization in relation to the literature and research objectives	N/A^c^

^a^TCBT: telephone-based cognitive behavioral theory.

^b^WBCT: web-based cognitive behavioral theory.

^c^N/A: not applicable.

An attempt was made to bracket the influence of the researcher’s prior knowledge and assumptions on the coding while acknowledging that this is never fully achievable and that the emerging analysis is necessarily a product of the interactions between interviewer, interviewee, and analyst, situated within their particular sociocultural, intellectual, and historical contexts. This analysis was guided by the research objectives, supervised by an investigator experienced in qualitative methods (FB) and led by junior nonclinical researchers trained in qualitative research but not CBT (SH, JH, and AF), one of whom (SH) was very familiar with the Regul8 intervention. They identified initial themes and subthemes, some of which resonated with the theoretical processes underpinning CBT. The initial and final themes and subthemes were reviewed and interpreted (SH, AF, JH, and AS) with input from trained CBT therapists (RM and TC), health psychologists (RM, TC, and FB), and an academic GP (HE). RM and TC led the development of the initial model underpinning the CBT intervention, and HE, SH, AS, and FB were involved in the development of Regul8. The credibility of this qualitative analysis was enhanced by the involvement of multiple researchers and the use of NVivo to facilitate (1) the iterative process of analysis moving between raw data, codes, and themes within a large data corpus and (2) systematic comparisons between and within individual participants.

## Results

### Overview

**Figure 1:**
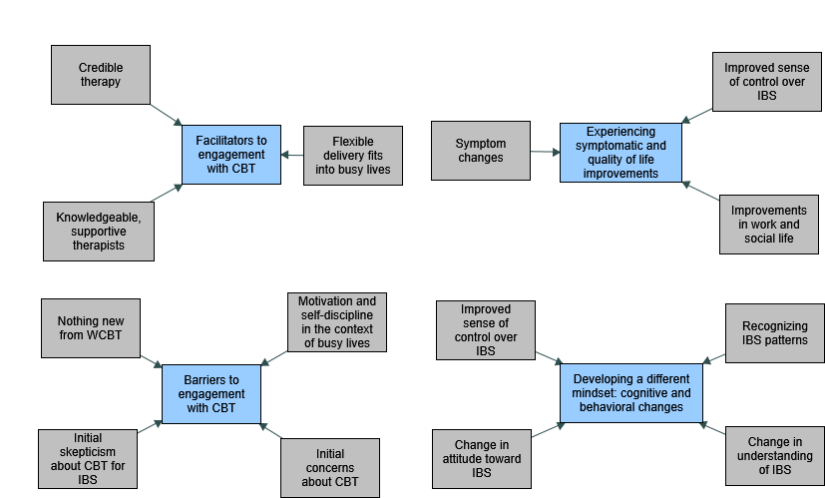
Summary of themes and subthemes capturing participant experiences of telephone and web-based cognitive behavioral theory for irritable bowel syndrome. CBT: cognitive behavioral theory; IBS: irritable bowel syndrome; WCBT: web-based cognitive behavioral theory.

A total of 4 main clusters of themes related to each objective were identified and were evident to some extent within both TCBT and WCBT groups: experiencing symptomatic and quality of life improvements; developing a different mindset: cognitive and behavioral changes; barriers to engagement with CBT; and facilitators to engagement with CBT. Each cluster comprised multiple themes, which are summarized in Figure 1. Below, we discuss these in detail, highlighting individual themes in italic typeface.

### Identifying Factors That Facilitate Engagement With Web-Delivered and Telephone-Delivered CBT for IBS

This objective was addressed by the themes collated under *facilitators to engagement with CBT*. High levels of satisfaction with CBT were suggested by participants’ positive comments about their experiences at both 3 months and 12 months and may have either facilitated or reflected high levels of engagement. Participants’ views of WCBT and TCBT being credible for IBS were shaped by perceptions that the ACTIB CBT took a systematic well-ordered approach; presented material in a professional, engaging, and accessible manner; and provided clear explanations of IBS and a convincing rationale for CBT. Early improvements in symptoms also contributed to a view of CBT being credible for IBS. This was true for both CBT groups, with no clear differences between the groups. A participant in the TCBT group explained:

Literally after the second week of doing it, sort of reading through the books and then—talking to [name of therapist] for the hour and going through everything, it was brilliant and the fact that it did really help, you know, week by week we were talking about different behaviours and—and I think, literally, I sort of saw improvement quite quickly really.

P24547, 3 months, TCBT

Similarly, a participant in the WCBT group reported:

...overall I was very, very pleased, it was nicely laid out, and I think that it’s really contributed into helping me overcome some of the issues that I’ve dealt with—I’ve been dealing with—up until that point.

P10074, 3 months, WCBT

Participants in both groups valued being able to talk to therapists who were perceived as knowledgeable and supportive. They valued:

just having someone to talk to

P39446, TCBT, 3 months

and found it particularly helpful to talk to someone who had IBS specific knowledge:

it’s just nice to have someone listen who is kind of—understands all the ins and outs

P45322, WCBT, 3 months

It would seem that the therapists were able to develop positive therapeutic relationships despite being constrained by the telephone to verbal communication only. Affective bonds were also evident, as participants described their therapists as follows:

really friendly

P25044, TCBT, 3 months

very nice to speak to

P20850, TCBT, 3 months

friendly and approachable

P16084, WCBT, 3 months

One potential drawback of web-based interventions, at least for some patients, is the lack of human interaction and support [12,13]. The telephone sessions provided to support the WCBT mitigated this risk, and participants described how this therapist contact helped to reinforce the messages from Regul8, provided the opportunity to discuss their particular case, and enabled them to ask questions and have them answered. In these ways, the 30-min telephone sessions helped to support engagement with the WCBT, in particular:

I think with the actual online sessions, they’ve been really helpful, but then, being able to talk... to my therapist, has really helped me kind of put them into practice. I think, for me, the online sessions themselves weren’t enough to deal with my case and my symptoms. I think being able to talk them over with the therapist has kind of—re-cemented things, reaffirmed things and just being able to talk about them has really helped.

P28570, WCBT, 3 months

talking to [therapist name] was really, really good. That was nice to have that kind of backup support and, you know, I had like—I think it was half an hour and it was a really good amount of time...

P45322, WCBT, 3 months

The flexibility afforded by both web-based delivery and telephone delivery was commented on positively by participants. Both modes of delivery were experienced as convenient for participants who were able to organize their own schedules for completing the web-based modules and did not have to physically travel to attend appointments:

I liked the way it was presented and it’s certainly helped me because I was able to do it my own time.

P16033, WCBT, 12 months

I liked that it was over the phone sometimes as well, that I didn’t always have to go somewhere and park and—that was good.

P25044, TCBT, 12 months

### Identifying Factors That Impede Engagement With Web-Delivered and Telephone-Delivered CBT for IBS

*Barriers to engagement with CBT* encompassed 4 main themes describing factors that impede engagement with WCBT and TCBT. Participants in both groups who discussed initial skepticism and initial concerns about the biopsychosocial nature of the intervention also reported mostly overcoming these potential barriers to engagement. Some described feeling skeptical about how remote CBT could be either relevant or effective for IBS, and this skepticism was typically couched in a perceived Cartesian disjunction between CBT as focused on mental processes and IBS as a physical condition. Skepticism in both groups was overcome through beginning the intervention and learning about the cognitive behavioral model of IBS used in ACTIB and developed in an earlier trial [25]. The cognitive behavioral model was presented by starting with the physiological and biological changes that underpin the IBS symptoms and then exploring how these changes can be influenced by thoughts, feelings, and behaviors as well as the autonomic nervous system responses linked to stress:

To be honest, when I started I was very skeptical, I couldn’t see how thought processes and things would actually affect your tummy, but when it’s explained through the literature and when you speak to a therapist, you can really see the connection between how you think and how your tummy reacts and—I think it just takes somebody to tell you...

P25119, TCBT, 3 months

Some participants across both groups expressed initial concerns about whether the telephone was an appropriate mode of delivery for CBT as a form of talking therapy. For a few (2 TCBT interviewees and 4 WCBT interviewees), these concerns persisted and appeared to derive from discomfort with the lack of nonverbal cues in telephone interactions:

obviously over the phone it’s slightly trickier than face-to-face. So we don’t know what the other person’s thinking or anything.

P40192, TCBT, 12 months

Others overcame their concerns once they had started telephone sessions and focused more on the content of the CBT being delivered:

I did think it is odd to do counselling over the phone, but now I think—actually—it doesn’t matter, it doesn’t really matter at all as long as the counselling is good.

P40210, TCBT, 3 months

Participants from both the TCBT and WCBT groups referred to the need for self-discipline and motivation to complete the homework tasks contained within the CBT program. Some found it difficult to motivate themselves to do this homework and may have been negatively affected by the connotations of this terminology:

I found it hard um… I’m not very good at doing homework and never have been and I don’t suppose I will be, um...so where it’s given my homework to do, I’ve not—I’ve not been, um let’s say a Grade A student.

P21339, WCBT, 3 months

Participants described wanting to do the homework to experience the anticipated health improvements but also reported finding it difficult to do this within the context of busy lives and competing priorities. There was a sense in both the WCBT and TCBT groups that investing more time in the program would result in getting more out of it:

I: What did you dislike about being in this group?

P: I think probably the discipline of having to do the homework... it’s kind of a bit of a paradox; I wanted to do the homework because I’m keen to participate and kind of make the best of it, but it’s kind of remembering to do it and—having something else to do during the week.

P25044, TCBT, 3 months

There could have been more I learned from it if I’d maybe done—spent more time on it or done it over a longer amount of time, then I might have got more out of it, but I did get a lot from it.

P40567, WCBT, 12 months

One final barrier that impeded engagement with WCBT (but was not identified in the narratives of TCBT participants) for some participants was the sense that this intervention did not offer anything new. This was mostly expressed by participants who felt that they had lived with their IBS for a long time and had already made themselves familiar with and tried to implement recommendations regarding lifestyle issues, including diet, stress, and physical activity. Such participants felt that the WCBT did not offer them any new insights or approaches to managing their IBS:

I’ve followed all the little sections on the trial, looking at your diet, looking at your stress, looking at your activity and I’ve kind of gone through all of those on my own in the last few, you know, over the years. So-from... my point of view, I didn’t get an awful lot out of it because it was already telling what I already knew.

P20066, WCBT, 3 months

Although this theme was only present in the WCBT arm, it is important to note that the content of the CBT program was the same in both TCBT and WCBT arms, with only the mode of delivery being different.

### Identifying Social and Psychological Processes of Change

Social and psychological processes of change were captured in the cluster of themes “Developing a different mindset: cognitive and behavioral changes.” Within this cluster, 4 themes described different changes experienced by participants across both WCBT and TCBT groups: changes in their understanding of IBS, changed attitudes toward IBS, a newfound ability to recognize IBS patterns, and subsequent changes in behavior associated with an increased sense of control over their IBS. At 3 months, participants in both groups felt they had an improved understanding of IBS as a reassuringly common condition that they could manage to some extent, based on their personal experience of improvements in IBS since commencing the trial:

I’m really pleased [with that] and it doesn’t mean the symptoms are completely gone, but it means that I understand them and I can control some of them. So I think that’s something I’m going to keep for life; it’s not something I’m going to forget about.

P40015, TCBT, 3 months

I feel relieved, relieved that—because going through the programme I realised that there are people out there who suffer exactly the same symptoms as I do, that it’s actually fairly common.

P10074, WCBT, 3 months

The CBT program taught participants to recognize their personal cognitive and behavioral patterns related to their IBS and enabled participants to evaluate their responses to these patterns. When these ideas resonated with individuals, they were able to reflect on them and think about things in a different way:

I think like with the thoughts, just being aware of the sort of things that can kind of perpetuate the cycle of stress. I think catastrophizing or black-and-white thinking and things like that, I think I can see them in myself and I think just being aware of that, you can kind of try and take a back step and see it in another way and re-evaluate the situation.

P26417, TCBT, 3 months

Possibly as a consequence of feeling that they understood their IBS better and could identify their personal patterns, participants also reported changing how they thought about IBS. For example, some described being able to feel more relaxed and less worried about their IBS. This change then appeared to be linked with positive changes in behavior, for example, enabling participants to liberate themselves from ingrained and socially limiting behaviors such as avoiding public or shared toilets:

I started thinking to myself, you know, I don’t need to stress and worry about it, there is a toilet here, it’s there if I can use it, which has helped, because I used to go home early from parties and things like that with a tummy ache. But now I can just think, you know, I’m working myself up about it and then, more often than not, the feeling goes away and then I’m absolutely fine.

P25119, TCBT, 3 months

Participants valued the tools and strategies that they developed through the CBT program when they were found to effectively help manage their (response to) IBS. In this way, both WCBT and TCBT appeared to promote an increased sense of control over IBS and greater self-efficacy for coping:

I would say I’m more in control than um previously because I have a whole series of tools to help me.

P20822, TCBT, 3 months

I feel so much more in control of my IBS and if something flares up I don’t feel like it’s the end of the world and I know that I’ve got strategies in place to be able to deal with them.

P28570, WCBT, 3 months

The cognitive and behavioral changes promoted by the CBT program were summed up by a participant in the TCBT group who experienced total relief from IBS, which was maintained at 12 months:

I now have a different mind-set, if you like, and a few little aids along the way, which help me to—remember the interviews and I don’t have any problems and haven’t had any problems since the ACTIB course finished.

P20822, TCBT, 12 months

### Insights Into the Quantitative Results of the ACTIB Trial

Cross-tabulating the themes by trial group and interview time point helped to relate the qualitative data to the following key findings from the quantitative trial: the overall effectiveness of WCBT and TCBT, the maintenance of effects at 12 months, and adherence to the interventions.

In the trial, all primary and secondary outcomes showed significant improvement in both CBT groups compared with TAU at 12 months [9] (the primary end point), and the overall pattern was for beneficial effects (in IBS symptom severity, mood, and impact on life roles) to be sustained, on average, from 3 to 6 to 12 months. There was also evidence of some sustained improvements and some attenuation of effects from 12 to 24 months [10]. The theme *Experiencing symptomatic and quality of life improvements* captures the range of patient-perceived benefits of the CBT program. The majority of participants in both therapy arms reported IBS symptom improvements over the period of the study; some participants also described how the symptomatic, cognitive, and behavioral changes associated with CBT contributed to improvements in their work and social lives. These improvements were particularly emphasized when participants were interviewed at 3 months but were still very evident in the 12-month interviews, demonstrating the lasting positive impact on the lives of these IBS patients:

I think it is much improved really; I’ve not had as much sort of constipation as what I used to have, so—so yes—for me, it has been really, really good.

P24547, TCBT, 3 months

if I do have a problem I know I can, you know, let my boss know and she’s fine. I’ll just say—I’m going to be in a bit late, whereas, you know, like I said before, I just—I would have just been like—I’m sick.

Pt 45322, WCBT, 3 months

I used to be very concerned about going round to people’s houses or going out to dinner—or going for food somewhere because I’d get very concerned that I might have a reaction and I’d need to run to the toilet straightaway, and I think that stress and worry beforehand would always then trigger a bout of IBS, but now it’s kind of—I started thinking to myself, you know, I don’t need to stress and worry about it, there is a toilet here, it’s there if I can use it, which has helped.

Pt 25119, TCBT, 3 months

However, while some participants maintained improvements and felt they would carry these on into the future, others felt they had not managed to maintain earlier improvements:

Unfortunately I’m unable to—control it as I did when I did the study and even though I still do a number of techniques and use the tools that I have learned last year, I’ve come to think that my mind has become immune to them and knows that these are just things I’m telling myself, but it’s not registering; so the mind controls my body, still.

P10074, WCBT, 12 months

To explore possible reasons why some participants felt they did not sustain beneficial changes after completion of active treatment, we classified participants as responders (n=22) and nonresponders (n=12) at 12 months and compared the themes and subthemes across these groups. A *responder* was defined as a participant with a 50-point improvement on the Irritable Bowel Syndrome Severity Scoring System from baseline to 12 months [8]. This analysis suggested that people classified as responders had more positive experiences of active treatment than those classified as nonresponders. At 3 months, the responders talked more about developing a different mindset and making cognitive and behavioral changes in response to CBT. Nonresponders placed more emphasis on barriers to engagement. This suggests that patients’ engagement with structured active therapy and their ability to embed cognitive and behavioral changes in their lives are, unsurprisingly, important for longer-term effects. Two case summaries presented in Textbox 1 help to illustrate this within the broader context of patients’ experiences. Participants’ reflections also suggest that any attenuation of beneficial effects could be partially mitigated by providing limited ongoing access to a therapist to help discourage relapse into unhelpful patterns:

I would have found it very useful, as I’m sure most participants would, to perhaps long-term, for this kind of treatment, have maybe somebody that you could contact, a point of contact from time to time, when you were having a particularly difficult time or needed to be reminded of something or to re-motivate you because like with all things, if we don’t have somebody behind us, I think we tend to have good intentions and then just go back to our old habits.

P20850, TCBT, 12 months

**Case summaries of responders and nonresponders to telephone-based cognitive behavioral therapy.**

Two participants were selected for in-depth presentation to illustrate how one responder and one nonresponder experienced cognitive behavioral therapy (CBT) for irritable bowel syndrome (IBS). These are not presented as representative cases but rather to showcase the interplay between themes as participants answered open-ended interview questions about their experiences over the course of a CBT trial. Both started the trial with *severe* IBS symptom severity scores, both were randomized to the telephone-based CBT arm, and both completed all of their telephone sessions including their booster calls. Participant P24547 maintained improvements at 12 months and was classified as a responder, whereas P40210 did not.

- Participant P24547

P24547 had experienced IBS symptoms for 15 years and did not really expect to benefit personally from the trial, having previously tried many treatments and “sort of thought it was something I just had to learn to live with, I didn*’*t think that I*’*d get a huge amount out of [the trial].” She had not previously tried CBT and did not express any reservations about it. When describing the nature of CBT and its impact on her IBS, P24547 emphasized the cognitive aspects of the treatment. For example, when asked about any changes experienced since starting the trial, she described how “I used to get quite stressed and worked up about—people at work. So [therapist name] was always saying that a lot of people with IBS have got sort of a level of perfection in themselves and I’ve definitely changed in the fact that I—I’m trying to do as much as I can, the best—but I don’t always—the best I can but I don’t always strive for perfection—which—which I think is that sort of side me has really helped, working through those exercises.” Related to this perceived need to make active changes to one’s thinking patterns, a strong sense of empowerment to make such changes and thus manage any symptoms emerged from P24547’s account of CBT. “I think through this therapy it really sort of highlighted some of the things, even simple things like doing more exercises and—making myself go out and—and not stay in and just sort of think and worry about my pain and get annoyed because of why this is happening to me, sort of thing. It was sort of understanding that there are things that I can do to work with the idea. So—for me—it was sort of being able to recognize the symptoms and then know how to deal with them.” During the trial, P24547 found the interactions with the therapist helped to motivate her to practice implementing her new cognitive and behavioral styles and was concerned that she might struggle to sustain these changes after completing the trial, but at 12 months explained how “actually I have continued to reflect on things and if I get cross about something, instead of getting myself wound up, which then tends to make my IBS even worse, I do—even literally last week—I got really cross about how something went and I then thought, no; I went to the toilet, I breathed, and I thought, right, how can I see this from their point of view, which is what the manual often went through. So even now I’m still finding it really useful.”

- Participant P40210

P40210 had experienced IBS symptoms for 24 years and was struggling with her IBS symptoms, feeling desperate for help at the start of the trial and “prepared to just try anything, I would have been happy to stand on my head if it had made it better.” She had “done CBT before for depression” and was a little apprehensive about delving into her emotions to start with but appreciated the new insights that she gained from CBT for IBS “now I know they [thoughts and feelings] are totally linked to my problems. So that*’*s been a positive thing that*’*s come out of it.” When asked about the effects of CBT, she evidenced her new insight into her IBS triggers but emphasized the improvements in her IBS symptoms and did not describe having adopted any cognitive or behavioral changes. “I have—what symptoms I*’*m having now ... are a huge amount less, I mean a massive amount less. So it*’*s mainly now wind, a bit of rumbling tummy and a little bit of—a little bit of acid reflux, a very small amount, but I can actually control that if I don*’*t eat certain things. And also—I have had one incident—a sort of very small amount of soiling incident, which is very unusual, I haven*’*t had that for about—three or four years. That was a day when I was particularly very stressed. And—small amounts of pain, you know, sort of spasm-type pain, sort of low down in my tummy, but really—a huge amount less than I had before.” P40210’s account of CBT did not suggest that she thought it was necessary to actively work on making cognitive changes to help her IBS. Instead, she seemed to engage with CBT on a more limited basis, accepting a new understanding of IBS but not acting on that understanding, instead focusing on dietary measures. Without cementing the underpinning cognitive changes, P40210 did not manage to sustain her dietary changes at 12 months: “But now a year later, I think I’ve probably fallen back into probably old habits really and I think a lot of that is to do with the fact that—and I was discussing this with my daughters earlier—the fact that my IBS problems have been going for a considerable length of time. So it’s as if the problems have outweighed the solutions, you know, the problems are more dominant than the solutions.”

Quantitative data suggested good adherence to TCBT and WCBT. In TCBT, 83.9% (156/186) of participants completed at least four telephone sessions, 88.1% (163/185) completed at least one telephone session, and 69.2% (128/185) completed at least four website sessions. Previous trials have varied in the way they have defined *adherence*, and reaching an appropriate definition is not straight forward [26]. We predefined *adherence* in our protocol and based it on the notion that participants would need to have received at least half of the program to be considered to have received CBT. Only 3 interviewees were classified as nonadherent in our trial, all of whom had received WCBT, and we were unable to identify any themes that differentiated adherent and nonadherent participants.

## Discussion

### Principal Findings

Participants provided positive feedback about both web-based and telephone-based CBT for IBS. They described improvements in IBS symptoms, positive changes in their understanding of and attitude toward IBS, and a newfound ability to recognize IBS patterns and change their own behaviors. This resulted in an increased feeling of control over their IBS and improved work and social life despite some initial skepticism regarding remote CBT for IBS. They highlighted the need for self-discipline to undertake CBT and maintain behavioral changes in the longer term but felt that the flexibility of telephone-based or web-based CBT and high-quality therapy input aided engagement. Telephone support in the WCBT group was important and valuable to the participants.

### Strengths and Limitations

This study was a rigorously conducted qualitative study that benefited from interviewing participants just after the CBT interventions to gather immediate perceptions at the end of the treatment (3 months) along with longer-term perceptions at 12 months. Participants were not interviewed again at 24 months, which reduced our ability to relate our qualitative findings to the 24-month quantitative follow-up [10]. The qualitative interviews enabled an exploration of individual differences in responding that were masked by the necessary focus in the trial data on group-level differences and changes over time. Participants were recruited from both primary and secondary care, which encompassed participants at different stages of their IBS journey, improving transferability to different settings. Participants interviewed had volunteered to participate, and all barring 3 were classified as *adherent* to the program. The results may have differed from a non–self-selecting, nonadherent sample. Participants were mostly White, British, and female, which is representative of the main ACTIB trial sample, but this sample does not allow us to draw inferences about how people from other demographic groups might experience CBT for IBS.

Although various measures were taken to minimize any inappropriate or uncritical influence of investigators’ preexisting frameworks, it is important to acknowledge that the interpretation of the data may have differed if conducted by a different research team. The measures taken to enhance analytic rigor included coding of data conducted by 3 different individuals, obtaining input throughout the analysis process from the multidisciplinary team, and consciously exploring possible alternate explanations and discussing them within the team. Furthermore, the individuals coding the data were not CBT therapists and did not consult the CBT therapists or model underpinning the intervention until the interpretation phase, after the subthemes had emerged.

### Comparison With Existing Literature

To the authors’ knowledge, this is the largest qualitative study to date exploring the experiences of participants undertaking web-based and telephone-based CBT for IBS. This study expands on work by Tonkin-Crine et al [13] by:

- including participants at 2 time points: 12 weeks and 12 months postbaseline, rather than just 12 weeks
- using a larger sample
- including participants who had received telephone-based CBT, rather than just web-based CBT

The results from this study showed similarities to the findings from the study by Tonkin-Crine et al [13], for example, the feelings of positivity about WCBT, and the need for self-motivation to carry them through the program. However, not all findings were replicated, notably, the current findings did not describe the website as *impersonal* or not personally *relevant enough*. This difference in findings may be accounted for by the different levels of telephone support in each study; WCBT participants in this study received three 30-min telephone sessions and two 30-min booster sessions, all with a trained CBT therapist. WCBT participants in the study by Tonkin-Crine et al [13] received much less telephone contact (1 session of 30-45 min), which was conducted by a practice nurse. Participants in this study valued the telephone support and the expert knowledge of the therapists, and it may be that this extra contact time with expertly trained therapists addressed these previously reported barriers. Indeed, having individuals providing support who are perceived as trustworthy, benevolent experts may be vital for such support to effectively engage patients in digital interventions [27].

### Conclusions and Implications for Future Research

Although acknowledging the difference in adherence rates between the TCBT and WCBT groups, more research is needed to investigate ways of increasing engagement in the web intervention. This may include a therapist portal where therapists can see what patients have completed on the web and provide support and encouragement online as well as by telephone. The data showed the telephone support in the WCBT group to be valued and important, and keeping this element in any future version would be beneficial.

Future versions of this CBT program may benefit from addressing the identified barrier around patients who have had IBS for a long-suffering feeling the web program *does not offer anything new*. It may be helpful for therapists to use Socratic questioning or guided discovery when discussing the patients’ personal cognitive behavioral model in the initial telephone call to unpick familiar and unfamiliar areas and, if appropriate, provide reassurance that more novel content will be covered later in the program.

Future CBT programs for IBS might benefit from addressing potential skepticism about the effectiveness of such treatment at the start of the program to help those with IBS understand how it might help. It is important to note that although this study focused on remote CBT for IBS, skepticism may be applicable more generally to CBT for IBS, rather than the delivery mode.

It is unclear why the theme around the website failing to *offer anything new* was only present in the WCBT arm; however, perhaps, the therapists in the TCBT arm were able to better personalize the content and focus on the parts most novel or applicable to the participants. Participants in the WCBT group indicated that the small amount of telephone support they received was helpful to keep them on track and provided an outlet to ask questions and talk about their progress. Future research may investigate the minimal amount of therapist contact time needed in the delivery of an effective web-based program for IBS, which may fall somewhere between 30 and 45 min of nurse contact offered in the previous trial [13] and the 150 min of therapist contact offered in the ACTIB trial. In addition, the longevity of this support and contact time needs to be explored, as some participants expressed the desire for longer-term support they could return to when they started to slip back into old habits. The potential source of this longer-term support also needs to be explored, for example, it is unclear whether this support would need to be provided by a therapist, a website, or a patient’s GP.

Both TCBT and WCBT for IBS were positively received by people with refractory IBS. The flexibility and perceived high quality of the interventions aided engagement. These forms of CBT have the potential to provide a lower-cost acceptable alternative to face-to-face CBT.

## Appendix

Multimedia Appendix 1 Three-month interview topic guide.

Multimedia Appendix 2 Twelve-month interview topic guide.

## Abbreviations

ACTIB: Assessing Cognitive behavioral Therapy in Irritable Bowel
CBT: cognitive behavioral theory
GP: general practitioner
IAPT: Improving Access to Psychological Therapies
IBS: irritable bowel syndrome
NHS: National Health Service
NIHR: National Institute for Health Research
TAU: treatment as usual
TCBT: telephone-based cognitive behavioral theory
WBCT: web-based cognitive behavioral theory
