# Supplementary material for: Patients’ Experiences of Telephone-Based and Web-Based Cognitive Behavioral Therapy for Irritable Bowel Syndrome: Longitudinal Qualitative Study
Source: J Med Internet Res. 2020 Nov 20;22(11):e18691. doi: 10.2196/18691 (PMC7718092; doi:10.2196/18691)
Supplement: Multimedia Appendix 2 [file jmir_v22i11e18691_app2.docx]

ACTIB: Interview Topic Guide (12 Month Interview)

**Introduction**

- Thank you for agreeing to take part in this interview [short hello].
- I would like to record the conversation we have today so that I can refer back to it at a later date. It enables me to listen to you better, is that ok?
- Before we start there are a few things I’d just like to mention.
- What we talk about will be used as part of the study, but anything said will remain anonymous. We’re going to ensure this by not using your real name when we type up the interview. Is that ok?
- If I ask a question that you don’t want to answer that is absolutely fine, just say so and I’ll ask you a different question. If at any point you would like to stop participating then please just tell me and we will stop the interview.
- I’m currently contacting a number of people who are taking part in the ACTIB study to find out about their experience of taking part in the trial, the treatments used in the trial, other IBS treatments they have tried in the past, and the way they feel.
- Anything you can tell me about your experiences including good and bad points would be useful.
- Do you have any questions before we start? Are you happy to continue?

**Section A. Firstly I would like to ask you about the trial you are taking part in.**

1. Now that it has been a year since you started in the trial, can you tell me what you think about your experiences with the study?
2. As part of the trial, you received [treatment as usual/website + telephone support/telephone CBT]. Now that it has been a little while since your treatment finished, could you tell me what you think about it?

Additional prompts to be used flexibly if necessary:

1. Can you tell me about anything that you feel changed because of this treatment? (Symptoms, Thoughts, Feelings, Lifestyle, Social/Relationships)
   1. Are there any other things that have changed?
2. How has your IBS been since we last spoke?
3. Do you think the treatments had any long-term effects on you?
4. Looking back on this treatment now....
   1. What do you think about this treatment for IBS?
   2. How do you feel about the use of this treatment for IBS?
   3. What do you like about this treatment?
   4. What do you dislike about this treatment?

**Section B. In this section of the interview, I’d like to find out about other treatments that you’ve tried for your IBS.**

1. Could you tell me about the most helpful treatments you have tried for your IBS since we last spoke?
   1. What did you like/dislike about these treatments?
2. Could you tell me about the least helpful treatments you have tried for your IBS since we last spoke?
   1. What did you like/dislike about these treatments?

For the treatment……………….

1. What led you to try out this treatment?
2. How would you compare these treatments to each other?
3. How do the past treatments compare with the treatment received during the trial?

**Section C. Until now, we have talked about your experience of taking part in the trial and the different treatments for your IBS. In the following section of the interview, I would like to find out more about how you feel in general and the way you manage your feelings. There are no right or wrong answers; I am just interested in your own personal experience.**

1. Could you talk me through how you feel on a typical day?
   1. Has it always been like this for you? Prompts: Have there been any changes recently? *If* *yes*, which ones?
2. How do you express your emotions or feelings to other people? Prompt: Why do you think this is? What about other people like colleagues of friends?
   1. Has it always been like this for you? Prompts: Have there been any changes recently? *If yes*, which ones?
3. Could you describe a time that you have experienced negative feelings?
   1. Can you tell me what you do/did when you experience negative feelings?
   2. Is there anything else that you do to cope with negative feelings? If yes, please describe.
4. Could you now describe a time that you have experienced positive feelings? Prompt: Can you tell me what you do when you experience positive feelings?

Additional prompts to be used flexibly if necessary:

1. How easy/difficult is to work out how you feel?
2. Do you feel in control of your emotions? Why do you think this is?

**Section D. In the final section of the interview, I’m interested in your thoughts about what happens after the trial.**

1. How have you been doing since you finished the first set of telephone support sessions? [for active arms only]
2. What do you think will happen next with your IBS?
3. Is there anything that we could do differently to improve our treatments for people with IBS?

**Closing and Ending**

- Thank you very much for sharing your thoughts and experiences with me today.
- What you’ve told me will really help us to understand patients’ experiences and hopefully to improve our treatments for IBS.
- Before we finish, is there anything else you want to tell me? Is there anything you want to ask me?
- Offer the participant a copy of the transcript and/or a summary of the findings.
